# Integrative missing value estimation for microarray data

**DOI:** 10.1186/1471-2105-7-449

**Published:** 2006-10-12

**Authors:** Jianjun Hu, Haifeng Li, Michael S Waterman, Xianghong Jasmine Zhou

**Affiliations:** 1 Molecular and Computational Biology Section, Department of Biological Sciences, University of Southern California, Los Angeles, CA, 900089, USA

## Abstract

**Background:**

Missing value estimation is an important preprocessing step in microarray analysis. Although several methods have been developed to solve this problem, their performance is unsatisfactory for datasets with high rates of missing data, high measurement noise, or limited numbers of samples. In fact, more than 80% of the time-series datasets in Stanford Microarray Database contain less than eight samples.

**Results:**

We present the integrative Missing Value Estimation method (iMISS) by incorporating information from multiple reference microarray datasets to improve missing value estimation. For each gene with missing data, we derive a consistent neighbor-gene list by taking reference data sets into consideration. To determine whether the given reference data sets are sufficiently informative for integration, we use a submatrix imputation approach. Our experiments showed that iMISS can significantly and consistently improve the accuracy of the state-of-the-art Local Least Square (LLS) imputation algorithm by up to 15% improvement in our benchmark tests.

**Conclusion:**

We demonstrated that the order-statistics-based integrative imputation algorithms can achieve significant improvements over the state-of-the-art missing value estimation approaches such as LLS and is especially good for imputing microarray datasets with a limited number of samples, high rates of missing data, or very noisy measurements. With the rapid accumulation of microarray datasets, the performance of our approach can be further improved by incorporating larger and more appropriate reference datasets.

## Background

Microarray technology has been one of the most useful tools in functional genomics research [1]. However, due to the inherent noise and the limitation of experimental systems, it is estimated that a microarray dataset on average has more than 5% missing values, affecting more than 60% of the genes [2]. Since many statistical analysis algorithms such as principal component analysis, singular value decomposition, support vector machines, and artificial neural networks either require complete datasets or are subject to significant performance degradations due to missing values [2], missing value estimation becomes an important preprocessing step for microarray data analysis.

The key issue of missing value estimation of microarray data is how to exploit the linear [3,4] or non-linear relationship [5] among the genes (rows) or the samples (columns). Since 2001, a series of microarray missing value estimation techniques have been developed [3,4,6-11]. These algorithms can be classified into three categories: global approaches, local approaches, and hybrid approaches which are the mixture of the previous two [12]. Global imputation algorithms such as singular value decomposition (SVDimpute) [6] and Bayesian principal components analysis (BPCA) [8] assume the existence of a covariance structure among all the genes or samples in the data matrix and thus are only suitable for datasets with strong global correlation, such as time-series datasets [8]. In addition, to achieve satisfactory performance, these global algorithms usually require a large number of samples (>20~30) as shown in [4] and [8]. However, many microarray datasets are non-time series or are noisy. For these types of datasets, local imputation algorithms such as K-nearest neighbor (KNN) [6], least square (LSImpute) [3], local least square (LLS) [4], collateral missing value estimation (CMVE) [9], and Gaussian mixture clustering (GMCImpute) are shown to be more suitable as they can exploit the dominant local similarity structure. These algorithms begin by selecting a set of genes with the highest relevance to the gene with missing values, based on Euclidian distance [4,6], Pearson's Correlation [3,4], or covariance estimate [9]. However, for noisy datasets or datasets with a limited number of samples, it is difficult to reliably identify true neighbor genes, especially when candidate genes contain missing values themselves. For example, LLS is not suggested to be used for datasets with no more than four samples and SVDimpute not for datasets with less than eight samples. In these cases, naturally, additional information should be exploited.

Recently, Oba *et al *[8] examined the effect of directly merging two microarray datasets from the same study for imputation, and found that this brought some improvement to BPCA and SVDimpute but degraded the performance of KNN. But they did not exploit external microarray datasets and no method was provided to select these external reference datasets. The first algorithm that explicitly utilizes external information is GOImpute proposed by Tuikkala et al. [13]. It exploits the functional similarity information embedded in the human-annotated GeneOntology (GO) databases – in addition to expression data similarity – to enhance the neighbor gene selection. For three datasets with less than 10 samples, GOImpute is shown to outperform KNN, but this is significant only when high rates of missing values exist so that KNN cannot estimate the neighborhood relationships correctly. GOImpute also failed to improve the LLS algorithm, which is one of the best local estimation algorithms. In addition, GOImpute is subject to the limitation on the number and accuracy of the gene functions annotated in gene ontology databases.

In this paper, we propose utilizing the rapidly accumulating microarray data in public databases to improve missing value estimation. Intuitively, if a set of genes frequently show expression similarity to the target gene over multiple data sets, they constitute a robust neighborhood which tend to show expression co-variations with the target gene. This is useful for imputing data sets with a few samples, for which insufficient information is available to select neighbor genes accurately. Here, we design a systematic framework of integrative Missing Value Estimation (iMISS) to automatically select appropriate reference microarray data sets, and discover consistent neighbor gene sets of a target gene based on order statistics. We show that our integrative approach can significantly and consistently improve the performance of the state-of-the-art LLS algorithm, which GOImpute did not achieve [13]. We compare the order-statistics-based integration method with a basic average-distance-based integration approach and show that the former is more robust. We applied iMISS to both LLS and KNN and demonstrate their performance differences due to their inherent natures.

## Results

### Data sets

We tested our algorithms on two groups of datasets downloaded from the public yeast microarray datasets [14]. The first group is composed of three datasets selected to represent diverse dataset types with the consideration that the iMISS approach is most useful for datasets with a small number samples. The first dataset (DER7) is a temporal gene expression dataset studying metabolic shift from fermentation to respiration in *Saccharomyces cerevisiae *[15]. The second dataset (OGA8) is on the study of phosphate accumulation and polyphosphate metabolism in *S. cerevisiae *by Ogawa et al [16]. The third dataset (FER4) is the on the study of gene expression patterns following adaptive evolution in yeast [17]. The second dataset group includes the alpha part (SP.ALPHA18) and the elutriation part (SP.ELU14) [18] of Spellman time series cell-cycle datasets. They have 18 and 14 samples respectively and are chosen for evaluating how the number of samples may influence the performance of the algorithms. Among these datasets, four have been used in previous missing value estimation studies: DER7, SP.ELU14, and OGA8 were used in GOImpute [13] and DER7, SP.ELU14, and SP.ALPHA18 were used in KNN imputation [6].

To create test datasets, we compiled a complete expression matrix from each of the above microarray datasets by removing any gene that contains missing values. Table 1 shows the size of the resulting test datasets. For each complete matrix, we can then generate arbitrary sets of test datasets with a given missing value rate and/or perturbation/noise level.

**Table 1:** Benchmark datasets

Name	No. of genes	No. of samples	Time-series	reference
DER7	5298	7	Yes	[15]
OGA8	5257	8	No	[16]
FER4	3685	4	No	[17]
ELU14	5192	14	Yes	[18]
ALPHA18	4053	18	Yes	[18]

We additionally collected 25 yeast cDNA microarray datasets each of which has less than 2% missing values (details see Supplementary website). When imputing a test dataset, the remaining 4 test datasets and the 25 additional datasets will serve as candidate reference datasets.

### Performance comparison with different neighbor size k

We compared the performance of the order-statistics-based integrative iLLS-O and iKNN-O to LLS and KNN methods. Although KNN has been shown to be inferior to most second-generation missing value estimation algorithms such as LLS, it is included here as a basis for comparison. We also compared the average-distance-based integrative approach with the order-statistics-based approach by running the same set of experiments with iLLS-D and iKNN-D. The six imputation algorithms are evaluated with different neighborhood sizes *k*. We used three datasets including DER7, OGA8, and FER4. For each dataset, 30 different sub-datasets are randomly generated according to a fixed missing value rate (5% unless otherwise specified). For the missing value rate of 5%, each entry of a target matrix will have a high probability (0.785) of being missing among the 30 simulations. We then apply all six algorithms and calculate the average and standard deviation of the normalized root mean square error (RMSE) relative to the zero-imputation method. For all integrative algorithms (iLLS-D, iKNN-D, iLLS-O, and iKNN-O), we use the dataset similarity score DS (with *T *= 500, see Methods) to automatically select the best five reference datasets from the 29 candidate reference datasets.

**Figure 2:**
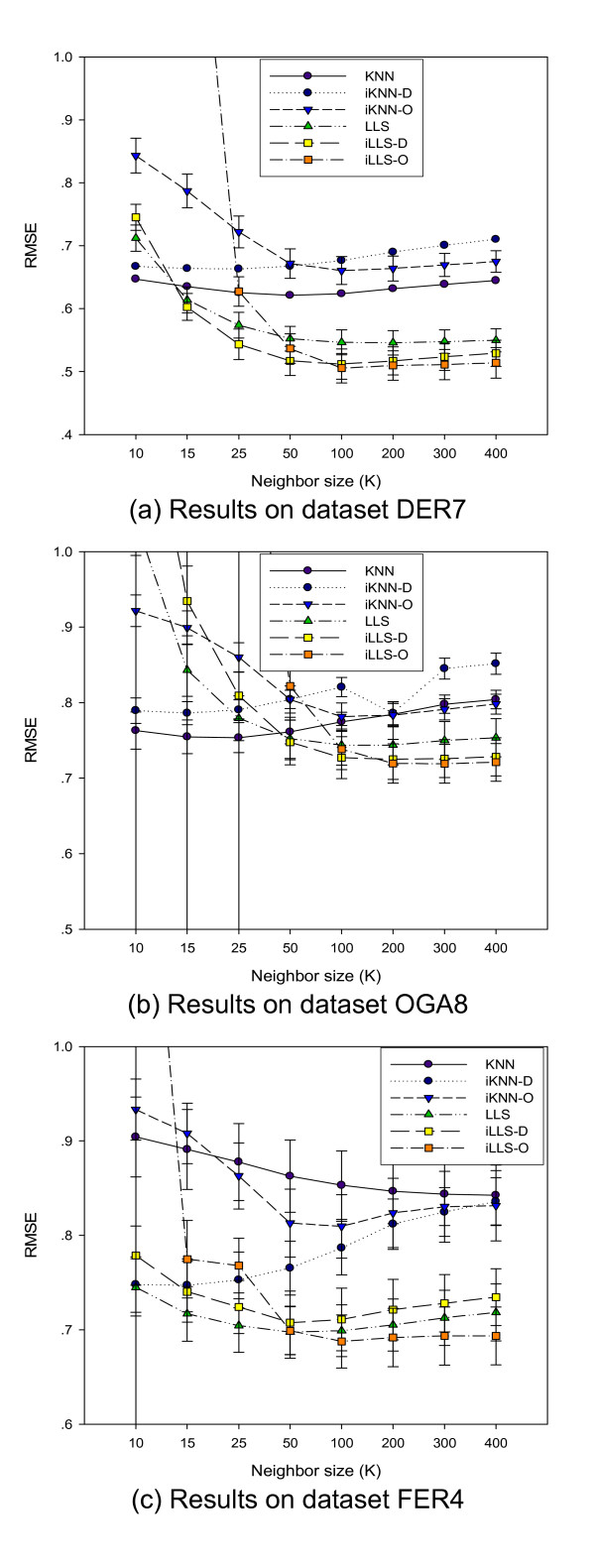
**Performances with respect to different neighbourhood size k**. LLS algorithm significantly outperforms KNN. The order-statistics-based iLLS-O is the best among all algorithms. Average-distance-based iLLS-D outperforms LLS in most cases.

Figure 2(a)–(c) show the performance of the algorithms on the three test datasets. Consistent with a previous report [4], the state-of-the-art LLS achieves much better performance than KNN for all the three datasets in their optimal range neighborhood size *k *(≥50 for LLS and between 10 and 25 for KNN). While it was previously reported that the GeneOntology-based GOImpute could not improve LLS further [13], our order-statistics-based iLLS-O method achieves significant improvement over LLS when *k *≥ 100 for the test datasets DER7, OGA8, and FER4 and is clearly the best algorithm overall. The maximal improvement is up to 14% reduction in terms of RMSE for the dataset OGA8. On the other hand, the average-distance-based method iLLS-D is inferior to the order-statistics-based method iLLS-O. While iLLS-D improves LLS for two of the three datasets, it leads to considerable performance degradation in the FER4 dataset, showing its inherent limitation due to its sensitivity to the variations of expression values across different reference datasets.

We also compared LLS based integrative algorithms iLLS-D and iLLS-O with KNN based algorithms iKNN-D and iKNN-O. While the integrative iLLS-O achieves consistent improvements over LLS for all three datasets, iKNN-O and iKNN-D outperforms KNN only in the dataset FER4 with four samples, showing KNN based integrative algorithms fail to achieve consistent performance gains. The difference in performance gain (over the base algorithms) between the integrative LLS and KNN methods can be attributed to the nature of these two algorithms: KNN uses the inverse of the gene expression distances as the *fixed *weights in weighting the contribution of neighbor genes, while LLS uses a regression procedure to determine the optimal weights for contributions of neighbor genes by taking into account their expression co-variation with the target gene. LLS derives an appropriate linear approximation for the missing values based on those neighbor genes that have strong linear relationships with the target gene. While selecting consistent neighbor genes based on average Euclidean distances across multiple datasets, some genes with similar absolute expression magnitudes as the target gene are more likely to be selected, despite having little or no expression correlation to the target gene. Such genes will receive high contribution weight in KNN and may affect estimation adversely, while LLS will dynamically assigning low or high contribution weights to them appropriately.

From the above three experiments, we found that on average the best performance of iLLS-D and iLLS-O are achieved with *k *around 100 to 200. Thus, in the following experiments, we use *k *= 150 for LLS, iLLS-D, and iLLS-O. Similarly for KNN, iKNN-D and iKNN-O, *k *will be set at 15 without loss of generality.

### Performance with respect to percentage of missing values

An important factor that influences accurate selection of neighbor genes is the missing value rate of the dataset. To evaluate how this factor may affect the performance of the imputation algorithms, we generate three series of benchmark datasets with missing value rates from 1% to 20% from DER7, OGA8, and FER4 and apply all six algorithms to them.

**Figure 3:**
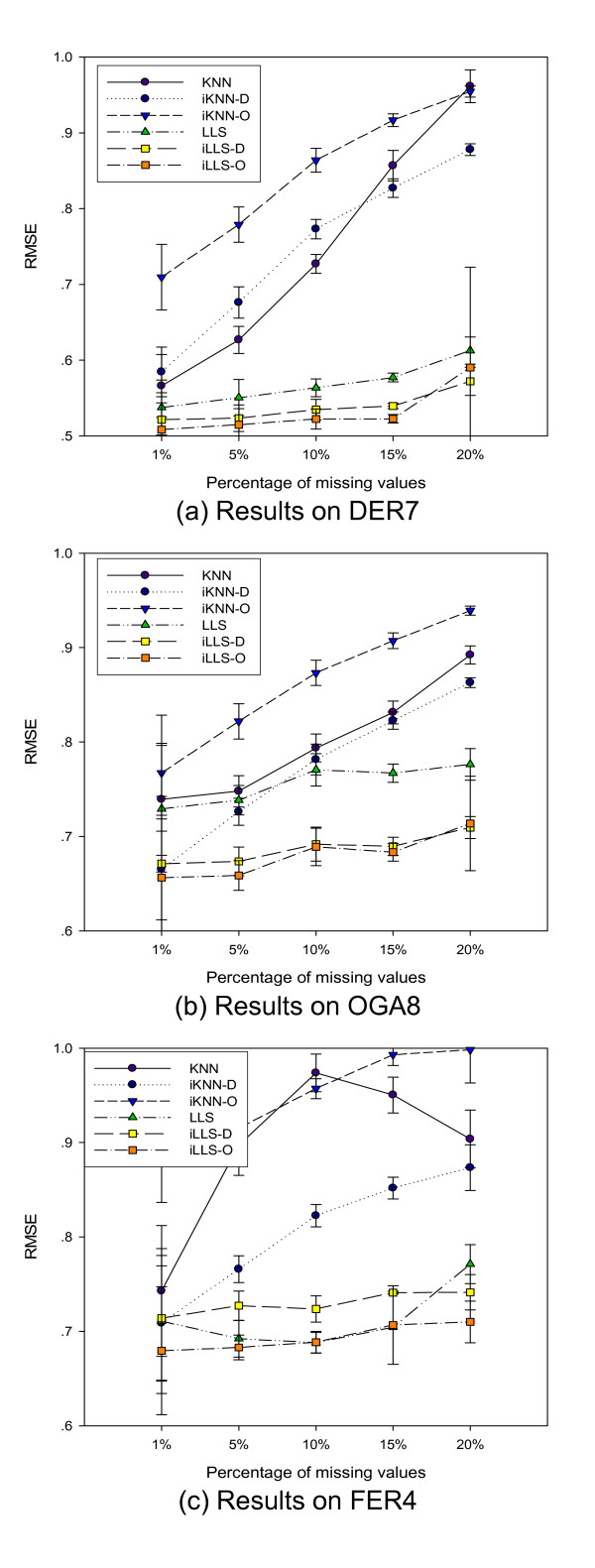
**Performance with respect to percentage of missing values**. Comparison of algorithm performance with respect to the percentage of missing values in the datasets. The iLLS-O algorithm achieves consistent gains across different percentages of missing values.

Figure 3(a)–(b) shows that the rate of missing values affects the performance of all algorithms for DER7, OGA8. Increasing missing value rate leads to significant performance degradations for KNN-based algorithms, but only moderate degradations for LLS-based algorithms. The consistent performance gains of integrative iLLS-D and iLLS-O over LLS with increasing missing value rates, demonstrates the benefits of the integrative approaches on datasets with high missing value rates. For KNN-based algorithms, the performance degradation rate for KNN with increasing missing value rate is greater than that for iKNN-D and iKNN-O, confirming that the integrative approaches do extract useful information from the reference datasets to increase the reliability of neighbor gene selection. We observed that the performance of the algorithms on FER4 with only four samples (Figure 3(c)) is quite different from that of DER7 and OGA8 where both KNN-D and KNN-O work worse than KNN. Here with only four samples, KNN-D algorithm performs consistently better than KNN across all missing value rates while iLLS-O is the dominating winner. We also found that surprisingly, for this dataset, KNN works better for FER4 test datasets with the missing value rate 20% than that with the missing value rate of 10%, showing that KNN is unable to pick appropriate neighbor genes for imputation if the number of samples is too small.

### Performance with respect to the number of samples

The number of samples in a dataset is another critical factor that influences the performance of imputation algorithms. For example, KNN is not suggested for use on datasets with less than four columns [6]. For local imputation algorithms, a small number of samples cannot provide sufficient information for reliable selection of neighbor genes. For global methods such as SVDimpute, too few columns lead to ill-conditioned matrices for singular value decomposition. To examine how the number of samples affects the integrative algorithms' performance, we picked the first *c *columns from the test datasets ELU14 and ALPHA18 with 14 and 18 samples respectively (*c *= 4, 5, 6, 10, 14 for ELU14 and *c *= 4, 5, 6, 10, 14, 18 for ALPHA18), and generated 30 benchmark datasets by randomly setting 5% of the values to be missing. We then tested all six algorithms on the benchmark datasets.

**Figure 4:**
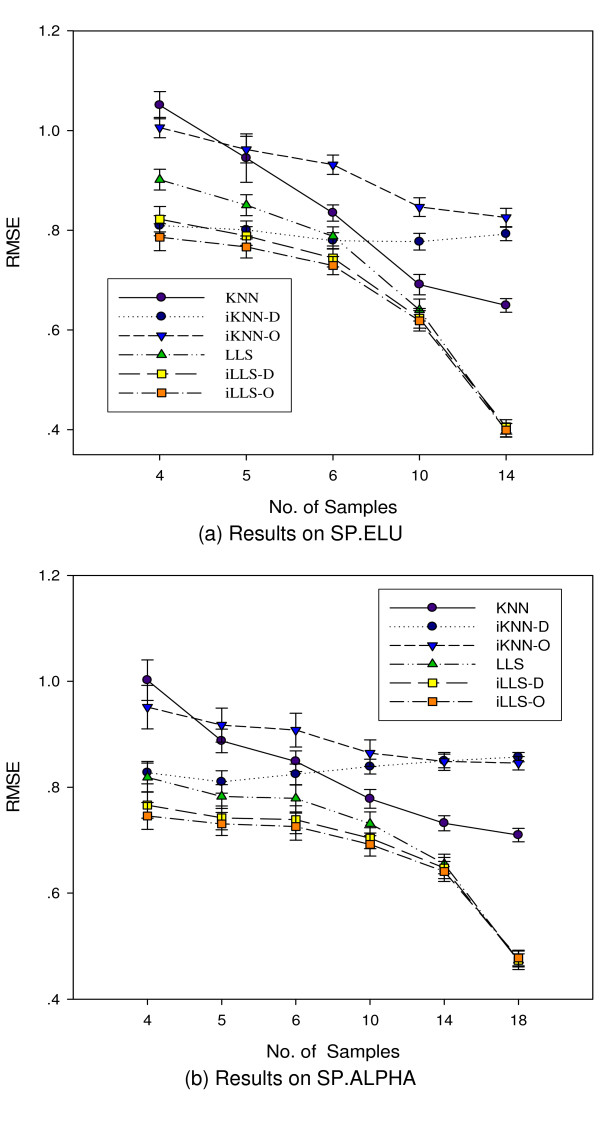
**Performance with respect to the number of samples**. Performance comparison with respect to the number of samples. The performance gain of iMISS algorithms is more significant when the number of samples in the microarray dataset is small.

Figure 4 shows how the performance of the algorithms changes with increasing number of samples in a dataset. A general trend is that both KNN and LLS are subject to significant performance degradation when too few samples are available. When the number of samples is less than 10, the integrative iLLS-D and iLLS-O consistently outperform LLS, and the performance gains increase with decreasing number of samples. For KNN, when the number of samples is no more than 6, the average-distance-based iKNN-D significantly outperforms KNN in both datasets. On the other hand, KNN works better than both iKNN-D and iKNN-O for both datasets when the number of samples is greater than six. When the number of samples drops to four, all integrative approaches including iLLS, iLLS-O, iKNN-D and iKNN-O outperform KNN or LLS respectively. These two experiments on time series datasets and the experiments on non-time series datasets OGA8 and DER7 as shown in Figure 2 showed that the proposed integrative missing value estimation approach is very useful for imputing datasets with a small number of samples which are insufficient for reliable estimation of good neighbor genes.

### Performance with respect to noise level

The performance of local imputation algorithms is also subject to measurement noise in microarray data. To evaluate the performance of the integrative algorithms for noisy datasets, we generate a set of benchmark datasets by adding Gaussian noise to the test datasets DER7, OGA8 and FER4. The magnitude of the perturbation is set in terms of standard deviations of Gaussian noise ranging from 0 to 0.25. For each benchmark dataset, we then generate 30 test datasets with randomly selected 5% missing values and applied the algorithms.

**Figure 5:**
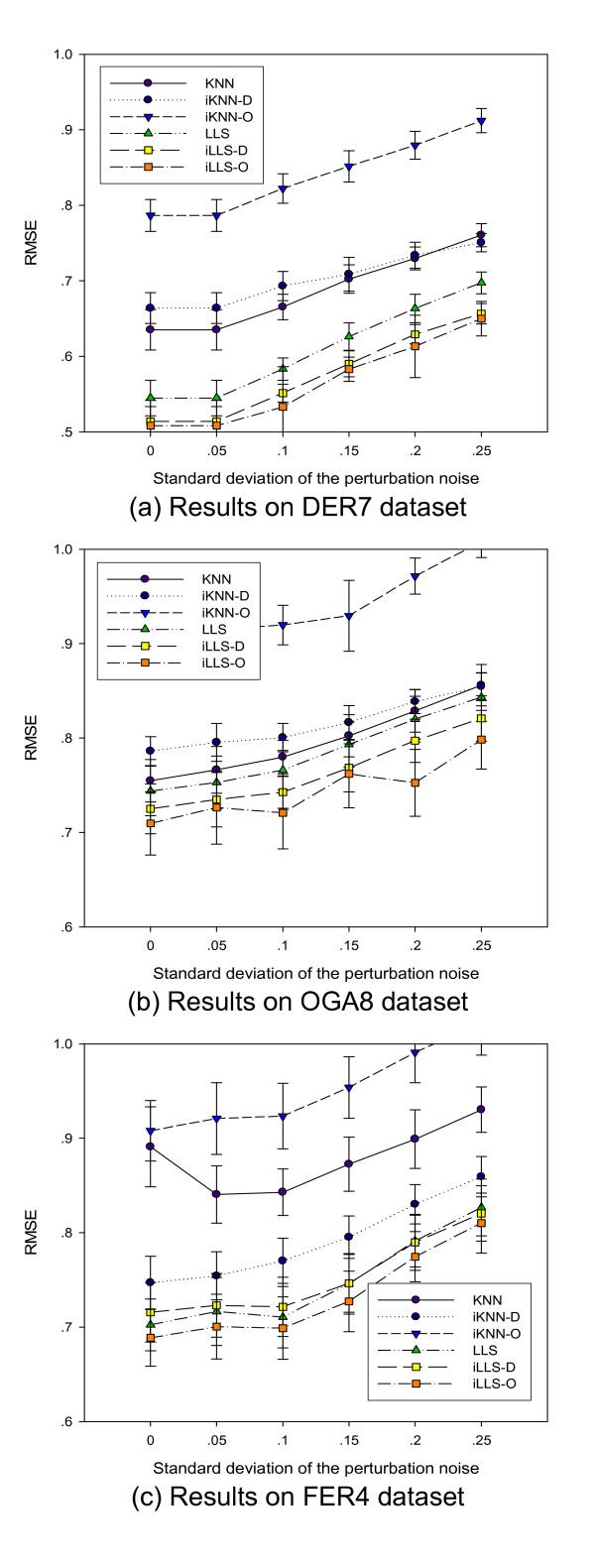
**Performance with respect to the noise levels**. Performance comparison with respect to the noise level of the dataset. The iLLS-O algorithm achieves consistent performance gain across all noise levels.

For datasets DER7 and OGA8, Figure 5(a,b) shows that the performance of all algorithms degrades with increasing noise levels, and iLLS-D and iLLS-O are consistently the best algorithms. Interestingly, the reduction of RMSE of iLLS-D and iLLS-O compared to LLS are consistent across all noise levels, showing the robustness of the integrative approaches against measurement noise. For KNN-based algorithms, when noise level increases, the performance gap between KNN and iKNN-O and iKNN-D decreases, showing that KNN is strongly subject to the noise levels in the data. This shows that the additional information from reference datasets makes the integrative algorithms more robust against measurement noise than the basic imputation algorithms. In the case of FER4 dataset (Figure 5c), the iKNN-D and iLLS-O consistently outperform KNN and LLS across all noise levels of the dataset.

### Performance with respect to the number of reference datasets

Another parameter which may influence the performance of the integrative imputation algorithm is the number of reference datasets. To examine this potential influence, we apply the algorithms to DER7, OGA8, and FER4 datasets with different numbers of reference datasets selected by the automatic dataset selection method (see Methods). We first calculated an ordered list of reference datasets with decreasing similarity to the target datasets, and then we selected the top 1 to 8 reference datasets to run the iMISS algorithms.

**Figure 6:**
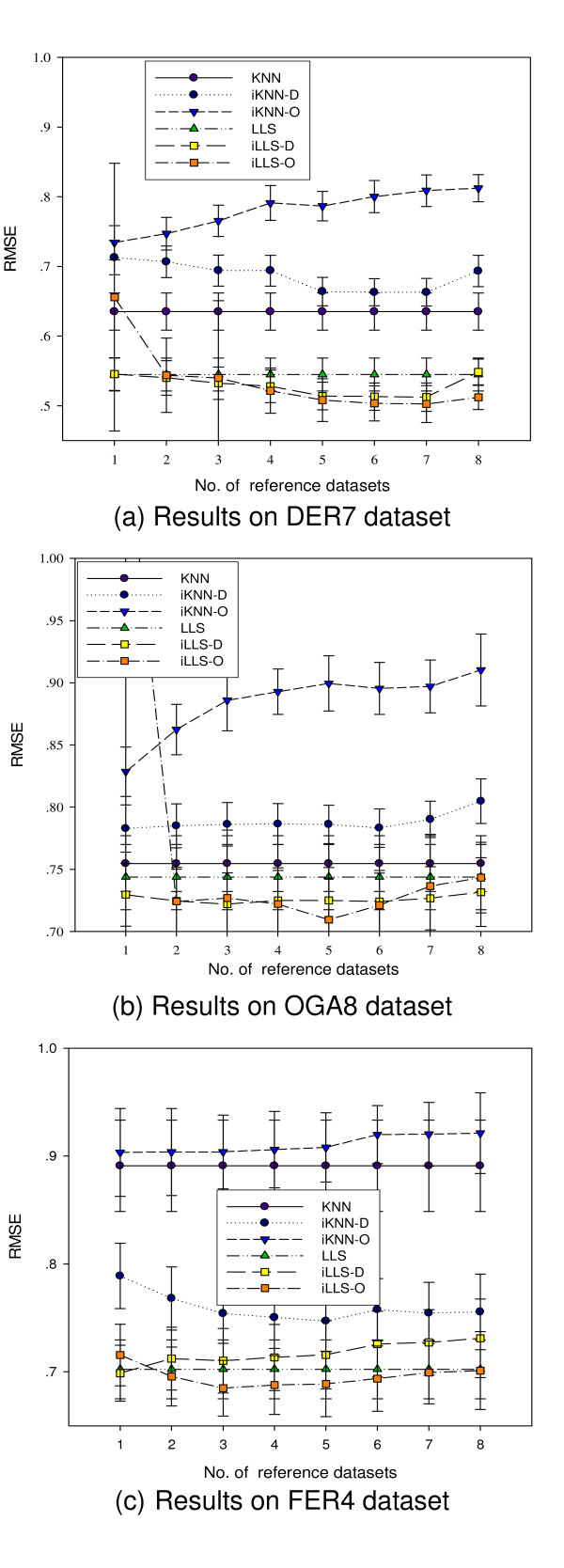
**Performance with respect to the number of reference datasets**. Performance comparison with respect to the number of reference datasets. In general, iMISS algorithms achieve best performance when the number of reference datasets is between 3–7.

Figure 6 shows two different trends of the algorithm performance with respect to the number of reference datasets. For integrative algorithms that outperform the base algorithms such as iLLS-O for all three datasets, iLLS-D for DER7 and OGA8 datasets, and iKNN-D for FER4 dataset, the performance of these integrative algorithms in general is not very sensitive to the number of reference datasets *R*. For example, the performance gains of iLLS-D and iLLS-O over LLS for DER7 and OGA8 are significant for *R *ranging from 4 to 7, although the optimal value of *R *depends on the target dataset. On the other hand, for integrative algorithms in which the neighbor selection method does not match well with the imputation procedure, increasing the number of reference datasets usually leads to even worse results. This is the case for KNN-O for all three datasets, KNN-D for OGA8, and iLLS-D for FER4. In both situations, including too many (e.g. eight in this study) reference datasets leads to performance degradation.

Although these results may depend on the matches between available reference datasets and the test datasets, we found that using five reference datasets is a reasonable choice in all our experiments, which we used in all other experiments in this study. Considering that usually only limited numbers of appropriate reference datasets are available and too many reference datasets (e.g. more than 10) can lead to long running time, we suggest the use of order statistics based imputation algorithms such as iKNN-O and iLLS-O only for up to seven reference datasets.

### Submatrix imputation method for choosing imputation algorithms

Given a microarray dataset to be imputed, we need to assess whether the available reference datasets are appropriate for the iMISS algorithms. For this purpose, we propose a submatrix imputation method and validate its usage as follows. First, we construct 40 benchmark datasets, ten from each of the complete matrices extracted from OGA8, DER7, FER4, and ELU14 by randomly setting 5% of the values to be missing. Next, we randomly pick one out of 30 benchmark datasets to generate a complete submatrix by removing any genes containing simulated missing values. From each submatrix we then generate 30 test datasets by again randomly setting 5% of the values to be missing. We term this class of datasets as *submatrix datasets*. Since the missing values in both benchmark data sets and the submatrix datasets are simulated, we can evaluate whether the performance of integrative algorithms on the two classes of datasets agrees well. If so, for any given microarray dataset to be imputed, we can use its submatrix to predict the performance of the integrative imputation algorithms on the original matrix.

As discussed in Methods Section, for each benchmark dataset of DER7, OGA8, FER4, and ELU14 we evaluated whether iKNN-D and iKNN-O (or iLLS-D and iLLS-O) work better than the basic algorithm KNN (or LLS). So there are four predictions of the relative performance for a benchmark dataset and the total prediction number is 16 for four benchmark datasets. We found that for 14 out of 16 cases the relative performance of the integrative algorithms with respect to KNN and LLS on the benchmark datasets and on the submatrix datasets is completely consistent. This is not unexpected as in most cases the submatrix datasets share most gene expression patterns with the original target dataset. If the reference datasets are not appropriate, the integrative algorithms will not work on the submatrix datasets and will not be suggested for imputing the original dataset. Similarly, when we want to select the most appropriate imputation algorithm for a given dataset, we can run all algorithms on the submatrices first and pick the one that works best as the final imputation algorithm to be used for the original dataset.

## Discussion

We have presented an integrative missing value estimation approach named iMISS for exploiting multiple external microarray datasets to improve local missing value estimation algorithms. By addressing the limitations of previous integration methods including the simple data merging method and GOImpute, we demonstrated that the order-statistics-based integrative imputation algorithms iLLS-O achieved significant improvements over the state-of-the-art LLS across a set of benchmark datasets. The average-distance-based iLLS-D is inferior to iLLS-O but also achieves considerable improvements over LLS. A comparative study on the integrative KNN shows that the popular KNN is less amenable to this extension due to its rigid weighted averaging procedure. Our analysis suggests that our integrative approach is especially beneficial for imputing datasets with a limited number of samples, high missing rates, or very noisy measurements. We proposed a dataset similarity scoring scheme to automatically select reference datasets given a target dataset. In addition, we also tested a singular value decomposition (SVD) approach to assess the similarity of two data matrices by comparing their corresponding eigen-spaces, which we found to be inferior to our proposed approach. We also proposed a submatrix imputation method to determine whether to use integrative imputation with a given collection of reference datasets. This method could as well be used to select the appropriate algorithm for imputing a given dataset, which would be very useful as many imputation algorithms exist and there is no absolute best one for all circumstances. As the performance of iMISS-based algorithms increases with better reference datasets, these algorithms are extensible as more microarray datasets become available in public databases. Our work suggests that missing value estimation of microarray data is different from pure statistical missing value estimation problems since the former has much more external information to exploit. It also shows that gene expression neighbourhood relationships are conserved in varying degrees among different microarray datasets and can be used to improve missing value estimation.

In this study, all data sets used were generated with the same cDNA microarray platform. It is known that even with the same platform technology, merging multiple microarray datasets directly is hindered by systematic variation among datasets, which is often beyond the capability of standard statistical normalization. Here, in the iMISS approach, since Euclidean distance calculation and subsequent neighbor gene ranking are performed within each dataset, ranked neighbor gene lists can be integrated across multiple datasets. However, when integrating microarray datasets from different platforms such as cDNA and Affymetrix platforms, the situation can be more complicated. Due to differences in the hybridization process, variation of expression magnitude of individual genes may bias distance estimation. In this situation, Pearson's correlation will be a more appropriate similarity measure to select neighbor genes, since it concerns relative gene expression changes instead of absolute values. However, using the cDNA datasets in this study, we found that the performance of using Pearson's correlation is inferior to that of Euclidean distance (data not shown). Thus, we recommend that users choose reference datasets from the same platform and use Euclidean distance as the similarity measure. With the rapid accumulation of microarray data, it is not a difficult task to collect significant number of datasets generated by the same technology platform.

The iMISS integration approach can be further improved in several aspects. For example, it is beneficial to include expert biological knowledge in the reference dataset selection process or as a screening step after the automatic selection. While we have used LLS as the base imputation algorithm to show the benefits of iMISS, other local imputation algorithms such as least squares (LSImpute) [3], local least squares (LLS) [4], collateral missing value estimation (CMVE) [9], and Gaussian mixture clustering (GMCImpute) can also be used within this integrative framework. Another potential enhancement is to differentiate the qualities of the reference datasets as a function of their number of samples, missing rates, or noise levels. In addition, in this study, we gave the same weight to the neighbor information derived from the target dataset and that derived from each reference dataset. Alternatively, users may weight the information from the target dataset higher based on their confidence in the data. Furthermore, neighbor gene information from different reference data sets may be weighted differently based on the similarity of the reference dataset to the target dataset. Another promising extension is to classify the available candidate datasets according to their experimental characteristics such as time-series, non-time-series, cancer, etc. For each category, we can convert multiple datasets into a gene expression neighborhood relationship database which can be used to improve imputation accuracy.

Our approach follows the integrative strategy for microarray analysis shown to be promising in previous studies [19-23]. This approach takes advantage of the conserved gene expression patterns among multiple datasets to enhance the signal/noise separation. In this generic framework, high-level information (e.g. the rank order statistics in this paper) is extracted from each dataset and combined to make more accurate decisions and predictions. This second-order-based approach makes feasible the integration of heterogeneous datasets, and its power will grow with the rapidly accumulation of public datasets.

## Methods

There are many ways to integrate gene-gene relationship information from external reference microarray datasets into existing imputation algorithms. The simplest approach is just to merge the samples from external data sets directly into the target dataset as is done by Oba *et al *[8]. However, systematic variation across data sets may bias the similarity estimation and thus the neighbor gene selection process. In the following, we propose and compare two methods, based on average distance and on rank order statistics respectively, to exploit the information in the reference microarray datasets to select neighbor genes reliably. We applied these two approaches to two local missing value imputation algorithms, the popular KNN [6] and the state-of-the-art LLS [4], both of which had been used by GOimpute [13].

### KNN and LLS Imputation

Assume a *M *× *N *matrix G=(gi,j)i,j=1M,N
 MathType@MTEF@5@5@+=feaafiart1ev1aqatCvAUfKttLearuWrP9MDH5MBPbIqV92AaeXatLxBI9gBaebbnrfifHhDYfgasaacH8akY=wiFfYdH8Gipec8Eeeu0xXdbba9frFj0=OqFfea0dXdd9vqai=hGuQ8kuc9pgc9s8qqaq=dirpe0xb9q8qiLsFr0=vr0=vr0dc8meaabaqaciaacaGaaeqabaqabeGadaaakeaacqWGhbWrcqGH9aqpcqGGOaakcqWGNbWzdaWgaaWcbaGaemyAaKMaeiilaWIaemOAaOgabeaakiabcMcaPmaaDaaaleaacqWGPbqAcqGGSaalcqWGQbGAcqGH9aqpcqaIXaqmaeaacqWGnbqtcqGGSaalcqWGobGtaaaaaa@3E84@ is the target microarray dataset with missing values to be imputed, where each row is the expression levels of a gene and each column represents a condition. In both KNN and LLS, for each gene with missing values (target gene), first we compute distances *d*_*i,j *_(e.g. Euclidian distances or Pearson's correlations) between candidate neighbor gene *G*_*j *_and the target gene *G*_*i *_in the target, in order to pick up the top *k *nearest neighbor genes. Specifically, to estimating the missing entry in the *l*th column of *G*_*i*_, KNN estimates the missing entry *G*_*i,l *_as the weighted average of neighboring genes: Gi,l=∑j=1kGj,l/di,j∑j=1k1/di,j(j≠i)
 MathType@MTEF@5@5@+=feaafiart1ev1aqatCvAUfKttLearuWrP9MDH5MBPbIqV92AaeXatLxBI9gBaebbnrfifHhDYfgasaacH8akY=wiFfYdH8Gipec8Eeeu0xXdbba9frFj0=OqFfea0dXdd9vqai=hGuQ8kuc9pgc9s8qqaq=dirpe0xb9q8qiLsFr0=vr0=vr0dc8meaabaqaciaacaGaaeqabaqabeGadaaakeaacqWGhbWrdaWgaaWcbaGaemyAaKMaeiilaWIaemiBaWgabeaakiabg2da9maalaaabaWaaabCaeaacqWGhbWrdaWgaaWcbaGaemOAaOMaeiilaWIaemiBaWgabeaakiabc+caViabdsgaKnaaBaaaleaacqWGPbqAcqGGSaalcqWGQbGAaeqaaaqaaiabdQgaQjabg2da9iabigdaXaqaaiabdUgaRbqdcqGHris5aaGcbaWaaabCaeaacqaIXaqmcqGGVaWlcqWGKbazdaWgaaWcbaGaemyAaKMaeiilaWIaemOAaOgabeaaaeaacqWGQbGAcqGH9aqpcqaIXaqmaeaacqWGRbWAa0GaeyyeIuoaaaGccqGGOaakcqWGQbGAcqGHGjsUcqWGPbqAcqGGPaqkaaa@5894@, where the *k *neighbor genes are those with the closest distances to the target gene. In contrast, LLS estimates all missing values of a gene simultaneously. After picking up the top *k *nearest neighbor genes, all the missing values of a gene/array u→
 MathType@MTEF@5@5@+=feaafiart1ev1aaatCvAUfKttLearuWrP9MDH5MBPbIqV92AaeXatLxBI9gBaebbnrfifHhDYfgasaacH8akY=wiFfYdH8Gipec8Eeeu0xXdbba9frFj0=OqFfea0dXdd9vqai=hGuQ8kuc9pgc9s8qqaq=dirpe0xb9q8qiLsFr0=vr0=vr0dc8meaabaqaciaacaGaaeqabaqabeGadaaakeaacuWG1bqDgaWcaaaa@2E31@ = (*α*_1_, *α*_2_,...,*α*_*n*_)^*T *^are estimated as a linear combination of values of other genes/arrays using standard least square regression:

u→
 MathType@MTEF@5@5@+=feaafiart1ev1aaatCvAUfKttLearuWrP9MDH5MBPbIqV92AaeXatLxBI9gBaebbnrfifHhDYfgasaacH8akY=wiFfYdH8Gipec8Eeeu0xXdbba9frFj0=OqFfea0dXdd9vqai=hGuQ8kuc9pgc9s8qqaq=dirpe0xb9q8qiLsFr0=vr0=vr0dc8meaabaqaciaacaGaaeqabaqabeGadaaakeaacuWG1bqDgaWcaaaa@2E31@^*T *^= *W*^*T*^*A*^†^*B*, where *W *is the known values of gene *G*_*i*_, *A*^† ^is the pseudo inverse of the sub-matrix composed of values of the *k *neighbor genes in the columns where *G*_*i *_has no missing values. *B *is the sub-matrix composed of values of the *k *neighbor genes in the columns where *G*_*i *_has missing values.

### Framework of integrative missing value estimation (iMISS)

**Figure 1:**
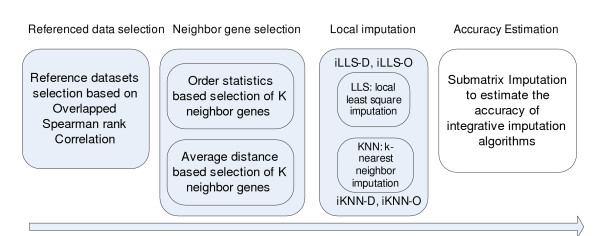
**Framework of iMISS**. iMISS (Integrative MISSing value estimation using multiple datasets) is composed four steps including reference dataset selection, neighbour gene selection, local imputation, and accuracy estimation.

In Figure 1 we show the framework of the integrative missing value estimation (iMISS). There are four steps in the estimation process. The first step is to select a set of microarray datasets as reference datasets based on their expression similarity to the target dataset. The second step is to select the top *k *neighbor genes based on the target dataset and the reference datasets. Two methods have been tested: one based on order statistics and the other on average distance. Next, one can use any local missing value estimation algorithm such as LLS and KNN to impute missing values in the dataset. Since it is difficult to know in advance whether the reference datasets are sufficient to produce high quality estimations, a fourth step is introduced to assess estimation quality of integrative imputation algorithms using a submatrix imputation approach.

### Selection of reference datasets

One important step in iMISS is the selection of a set of appropriate reference datasets that show similar gene expression patterns with the target dataset to be imputed. To ensure that only reliable gene relationship information is used, we remove datasets that have more than 2% missing values. We also remove those datasets that have too few samples (less than 5). From the remaining microarray datasets, there are two basic ways to select reference data sets. First, one can utilize biological knowledge. For example, it is reasonable to use cell-cycle datasets to impute a cell-cycle dataset. However, since there are tens or even hundreds of potential reference candidate datasets available, examining their experiment contexts is tedious. Here we propose an automatic method for reference dataset selection by measuring dataset similarity.

For each gene *G*_*i *_in the target dataset, we pick up the top *T *genes with smallest Euclidean distances to *G*_*i *_in the target dataset *D*_0 _and in a potential reference dataset *D*_*k*_. We then calculate the percentage *ρ*_*i *_of the overlapping genes out of these two sets of *T *genes. To further incorporate the rank order information of the overlapping genes in terms of their distances to *G*_*i*_, we further calculate the Spearman's rank correlation coefficient *r*_*i *_for the overlapping genes. We define the dataset similarity (DS) score as

DS=1|GI|∑i=1|GI|ρi⋅ri,
 MathType@MTEF@5@5@+=feaafiart1ev1aqatCvAUfKttLearuWrP9MDH5MBPbIqV92AaeXatLxBI9gBaebbnrfifHhDYfgasaacH8akY=wiFfYdH8Gipec8Eeeu0xXdbba9frFj0=OqFfea0dXdd9vqai=hGuQ8kuc9pgc9s8qqaq=dirpe0xb9q8qiLsFr0=vr0=vr0dc8meaabaqaciaacaGaaeqabaqabeGadaaakeaacqWGebarcqWGtbWucqGH9aqpdaWcaaqaaiabigdaXaqaaiabcYha8jabdEeahnaaBaaaleaacqWGjbqsaeqaaOGaeiiFaWhaamaaqahabaacciGae8xWdi3aaSbaaSqaaiabdMgaPbqabaaabaGaemyAaKMaeyypa0JaeGymaedabaGaeiiFaWNaem4raC0aaSbaaWqaaiabdMeajbqabaWccqGG8baFa0GaeyyeIuoakiabgwSixlabdkhaYnaaBaaaleaacqWGPbqAaeqaaOGaeiilaWcaaa@4ACD@

where |*G*_*I*_| is the number of common genes in the datasets *D*_0 _and *D*_*k*_.

The motivation for the above scoring scheme is that since only top *T *(usually ≤500) genes are used in local imputation algorithms, the score shall reflect the similarity of gene neighbor structures in two data sets, i.e. the number of shared neighbor genes and the rank order of those neighbor genes.

### Integration using average distance

This approach is introduced as a comparison to the proposed order-statistics-based integration approach. Specifically, the expression distance or similarity of two genes is calculated as the average of the normalized distances or similarities (normalized by the number of columns) across all reference datasets and the target dataset. Assuming Euclidian distance is used, for a target gene with missing values, we first calculate the normalized distances di,jk
 MathType@MTEF@5@5@+=feaafiart1ev1aaatCvAUfKttLearuWrP9MDH5MBPbIqV92AaeXatLxBI9gBaebbnrfifHhDYfgasaacH8akY=wiFfYdH8Gipec8Eeeu0xXdbba9frFj0=OqFfea0dXdd9vqai=hGuQ8kuc9pgc9s8qqaq=dirpe0xb9q8qiLsFr0=vr0=vr0dc8meaabaqaciaacaGaaeqabaqabeGadaaakeaacqWGKbazdaqhaaWcbaGaemyAaKMaeiilaWIaemOAaOgabaGaem4AaSgaaaaa@3321@ between the target gene *G*_*i *_and all remaining genes *G*_*j*_, *j *≠ *i*, *j *= 0,...,|*D*_0_| in both the target dataset and all reference datasets *D*_*k*_, *k *= 0, 1,...,*n*:

di,jk=∑l=1N(xi,l−xj,l)2/N
 MathType@MTEF@5@5@+=feaafiart1ev1aaatCvAUfKttLearuWrP9MDH5MBPbIqV92AaeXatLxBI9gBaebbnrfifHhDYfgasaacH8akY=wiFfYdH8Gipec8Eeeu0xXdbba9frFj0=OqFfea0dXdd9vqai=hGuQ8kuc9pgc9s8qqaq=dirpe0xb9q8qiLsFr0=vr0=vr0dc8meaabaqaciaacaGaaeqabaqabeGadaaakeaacqWGKbazdaqhaaWcbaGaemyAaKMaeiilaWIaemOAaOgabaGaem4AaSgaaOGaeyypa0ZaaOaaaeaadaaeWbqaaiabcIcaOiabdIha4naaBaaaleaacqWGPbqAcqGGSaalcqWGSbaBaeqaaOGaeyOeI0IaemiEaG3aaSbaaSqaaiabdQgaQjabcYcaSiabdYgaSbqabaGccqGGPaqkdaahaaWcbeqaaiabikdaYaaaaeaacqWGSbaBcqGH9aqpcqaIXaqmaeaacqWGobGta0GaeyyeIuoakiabc+caViabd6eaobWcbeaaaaa@4B6A@, where *x*_*i,l *_and *x*_*j,l *_are the valid expression values of genes *G*_*i *_and *G*_*j*_; and *N *is the total number of samples that have valid expression values for these two genes in the data set *D*_*k*_.

We then define the distance of a candidate neighbor gene to the target gene as the average of the normalized distances from all datasets and use it to select neighbor genes.

di,j=(∑k=0ndi,jk)/(n+1)
 MathType@MTEF@5@5@+=feaafiart1ev1aaatCvAUfKttLearuWrP9MDH5MBPbIqV92AaeXatLxBI9gBaebbnrfifHhDYfgasaacH8akY=wiFfYdH8Gipec8Eeeu0xXdbba9frFj0=OqFfea0dXdd9vqai=hGuQ8kuc9pgc9s8qqaq=dirpe0xb9q8qiLsFr0=vr0=vr0dc8meaabaqaciaacaGaaeqabaqabeGadaaakeaacqWGKbazdaWgaaWcbaGaemyAaKMaeiilaWIaemOAaOgabeaakiabg2da9iabcIcaOmaaqahabaGaemizaq2aa0baaSqaaiabdMgaPjabcYcaSiabdQgaQbqaaiabdUgaRbaaaeaacqWGRbWAcqGH9aqpcqaIWaamaeaacqWGUbGBa0GaeyyeIuoakiabcMcaPiabc+caViabcIcaOiabd6gaUjabgUcaRiabigdaXiabcMcaPaaa@47C0@

### Integration using order statistics

Average-distance-based integration is sensitive to variations or outliers of the expression values across the target and the reference datasets. Here we propose an order statistics measurement to select consistent neighbor genes across multiple datasets by integrating the distance rank order of genes to the target gene in all data sets. This approach is more robust since variations of the expression values that do not change the relative ranks will not affect the selection of neighbor genes. Order statistics has been used to measure the significance of gene-gene correlation across multiple species [24]. Here we use it to select *k *genes that are consistently ranked as neighbor genes across multiple datasets.

Intuitively, in the case of a single dataset, both KNN and LLS methods rank candidate neighbor genes based on their distances to the target gene in the target dataset and then pick up the top *k *genes with shortest distances. Similarly, with multiple datasets, we can rank the candidate genes in both the target and each of the reference datasets. To select genes that are consistently ranked high in all datasets, we calculate the probability (P-value) of observing their ranks in all datasets. The complete procedure is as follows. First, for each gene *G*_*i*_, *i *= 1,...,|*D*_0_|, where |*D*_0_| is the number of genes in the target dataset *D*_0_, we rank the set of candidate neighbor genes *G*_*j*_, *j *≠ *i*, *j *= 1,...,|*D*_0_| according to their distances to the target gene in the target dataset. Since different datasets may contain different number of genes, the ranks are then divided by the number (|*D*_0_| - 1) of candidate genes to get the rank ratio *r*_*j*,0 _of the gene *G*_*j *_in the target dataset. Then for each reference dataset (*D*_*k*_, *k *= 1, 2,...,*n*), we first check if *G*_*i *_exists in *D*_*k*_, if it does not, then we set rank ratio *r*_*j,k *_as unavailable. We then determine the intersection set of the *N*_*k *_genes that exist in both the target dataset *D*_0 _and the reference dataset *D*_*k*_. These genes are then ranked according to their distances to the target gene *G*_*i *_in *D*_*k *_and their rank ratios *r*_*j,k *_can be calculated by dividing their ranks (ranging from 1 to *N*_*k*_) by *N*_*k*_.

Assuming the rank ratios *r*_*j,k*_, *k *= 0,...,*n *for a gene *G*_*i *_are drawn independently and uniformly, the *P*-value from the joint cumulative distribution of the *n*-dimensional order statistics can be written as:

*P*(*r*_*j*,0_, *r*_*j*,1_, *r*_*j*,2_,...,*r*_*j,n*_) = n!∫0rj,0∫s0rj,1∫s1rj,2...∫sn−1rj,nds0ds1ds2...dsn
 MathType@MTEF@5@5@+=feaafiart1ev1aaatCvAUfKttLearuWrP9MDH5MBPbIqV92AaeXatLxBI9gBaebbnrfifHhDYfgasaacH8akY=wiFfYdH8Gipec8Eeeu0xXdbba9frFj0=OqFfea0dXdd9vqai=hGuQ8kuc9pgc9s8qqaq=dirpe0xb9q8qiLsFr0=vr0=vr0dc8meaabaqaciaacaGaaeqabaqabeGadaaakeaacqWGUbGBcqGGHaqidaWdXaqaamaapedabaWaa8qmaeaacqGGUaGlcqGGUaGlcqGGUaGldaWdXaqaaiabdsgaKjabdohaZnaaBaaaleaacqaIWaamaeqaaOGaemizaqMaem4Cam3aaSbaaSqaaiabigdaXaqabaGccqWGKbazcqWGZbWCdaWgaaWcbaGaeGOmaidabeaakiabc6caUiabc6caUiabc6caUiabdsgaKjabdohaZnaaBaaaleaacqWGUbGBaeqaaaqaaiabdohaZnaaBaaameaacqWGUbGBcqGHsislcqaIXaqmaeqaaaWcbaGaemOCai3aaSbaaWqaaiabdQgaQjabcYcaSiabd6gaUbqabaaaniabgUIiYdaaleaacqWGZbWCdaWgaaadbaGaeGymaedabeaaaSqaaiabdkhaYnaaBaaameaacqWGQbGAcqGGSaalcqaIYaGmaeqaaaqdcqGHRiI8aaWcbaGaem4Cam3aaSbaaWqaaiabicdaWaqabaaaleaacqWGYbGCdaWgaaadbaGaemOAaOMaeiilaWIaeGymaedabeaaa0Gaey4kIipaaSqaaiabicdaWaqaaiabdkhaYnaaBaaameaacqWGQbGAcqGGSaalcqaIWaamaeqaaaqdcqGHRiI8aaaa@6B63@ where *s*_0_, *s*_1_,...,*s*_*n *_are integral variables.

The above *P*-value can be computed using a recursive formula:

*P*(*r*_*j*,0_, *r*_*j*,1_,...,*r*_*j,n*_) = ∑j=0n(rj,n−j+1−rj,n−j)P(rj,0,rj,1,...,rj,n−j,rj,n−j+2,...,rj,n)
 MathType@MTEF@5@5@+=feaafiart1ev1aaatCvAUfKttLearuWrP9MDH5MBPbIqV92AaeXatLxBI9gBaebbnrfifHhDYfgasaacH8akY=wiFfYdH8Gipec8Eeeu0xXdbba9frFj0=OqFfea0dXdd9vqai=hGuQ8kuc9pgc9s8qqaq=dirpe0xb9q8qiLsFr0=vr0=vr0dc8meaabaqaciaacaGaaeqabaqabeGadaaakeaadaaeWbqaaiabcIcaOiabdkhaYnaaBaaaleaacqWGQbGAcqGGSaalcqWGUbGBcqGHsislcqWGQbGAcqGHRaWkcqaIXaqmaeqaaOGaeyOeI0IaemOCai3aaSbaaSqaaiabdQgaQjabcYcaSiabd6gaUjabgkHiTiabdQgaQbqabaGccqGGPaqkcqWGqbaucqGGOaakcqWGYbGCdaWgaaWcbaGaemOAaOMaeiilaWIaeGimaadabeaakiabcYcaSiabdkhaYnaaBaaaleaacqWGQbGAcqGGSaalcqaIXaqmaeqaaOGaeiilaWIaeiOla4IaeiOla4IaeiOla4IaeiilaWIaemOCai3aaSbaaSqaaiabdQgaQjabcYcaSiabd6gaUjabgkHiTiabdQgaQbqabaGccqGGSaalcqWGYbGCdaWgaaWcbaGaemOAaOMaeiilaWIaemOBa4MaeyOeI0IaemOAaOMaey4kaSIaeGOmaidabeaakiabcYcaSiabc6caUiabc6caUiabc6caUiabcYcaSiabdkhaYnaaBaaaleaacqWGQbGAcqGGSaalcqWGUbGBaeqaaaqaaiabdQgaQjabg2da9iabicdaWaqaaiabd6gaUbqdcqGHris5aOGaeiykaKcaaa@7470@ where *r*_*j*,0 _= 0

So for each gene *G*_*i*_, we can calculate the *P*-value of rank ratios for all candidate neighbor genes and then pick the top *k *genes with lowest P-value as its consistent neighbors. The order-statistics-based iMISS algorithms derived from KNN and LLS are denoted as iKNN-O and iLLS-O, respectively. The imputation algorithms derived by applying this average-distance-based integration method to KNN and LLS are termed iKNN-D and iLLS-D, respectively.

### Submatrix imputation method to assess appropriateness of integrative algorithm

The performance of iMISS-based integrative imputation algorithms depends on the quality of the reference microarray datasets and their similarity to the target dataset. However, there is no absolute dataset similarity threshold that can guarantee the quality of estimation. Here we propose a submatrix imputation procedure to determine whether our integrative approach can be advantageous to traditional approaches given a set of potential reference datasets.

The submatrix imputation method predicts the performance of a given imputation algorithm by evaluating its performance on a set of evaluation datasets generated from a complete submatrix extracted from the target dataset. First, from the target dataset we remove all genes with missing values to generate a complete submatrix. Next, we generate 30 evaluation datasets from the complete matrix by removing 5% of the values randomly. We then run all the imputation algorithms to be compared on the 30 evaluation datasets. Now we can evaluate the quality of estimation for each algorithm since we know the "true" values of the missing values. To assess the significance of the performance difference between two algorithms A and B, we use student's *t*-test to compare their RMSE errors on the 30 evaluation datasets. If the p-value is less than 0.05 and the mean RMSE of A is smaller than B, then the two algorithms are regarded to show different performance and A is better than B. By comparing iLLS-D, iLLS-O (or iKNN-D and iKNN-O) with the original LLS (or KNN) algorithm, we can determine whether the use of integrative algorithms is appropriate.

### Evaluation methods

There are two standard metrics to evaluate the prediction accuracy given the true values of the missing entries. One is normalized RMS error, defined as the root mean squared error between the imputed matrix and the original matrix normalized by the average data value in the complete dataset [4,6,8]. The other metric is RMSE [11], the root mean squared error between the true values and the imputed values divided by the root mean squared true values of the missing entries:

RMSE=mean[y→imputed−y→true]2/mean[y→true]2     (2)
 MathType@MTEF@5@5@+=feaafiart1ev1aaatCvAUfKttLearuWrP9MDH5MBPbIqV92AaeXatLxBI9gBaebbnrfifHhDYfgasaacH8akY=wiFfYdH8Gipec8Eeeu0xXdbba9frFj0=OqFfea0dXdd9vqai=hGuQ8kuc9pgc9s8qqaq=dirpe0xb9q8qiLsFr0=vr0=vr0dc8meaabaqaciaacaGaaeqabaqabeGadaaakeaacqqGsbGucqqGnbqtcqqGtbWucqqGfbqrcqGH9aqpdaGcaaqaaiabd2gaTjabdwgaLjabdggaHjabd6gaUjabcUfaBjqbdMha5zaalaWaaSbaaSqaaiabdMgaPjabd2gaTjabdchaWjabdwha1jabdsha0jabdwgaLjabdsgaKbqabaGccqGHsislcuWG5bqEgaWcamaaBaaaleaacqWG0baDcqWGYbGCcqWG1bqDcqWGLbqzaeqaaOGaeiyxa01aaWbaaSqabeaacqaIYaGmaaaabeaakiabc+caVmaakaaabaGaemyBa0MaemyzauMaemyyaeMaemOBa4Maei4waSLafmyEaKNbaSaadaWgaaWcbaGaemiDaqNaemOCaiNaemyDauNaemyzaugabeaakiabc2faDnaaCaaaleqabaGaeGOmaidaaaqabaGccaWLjaGaaCzcamaabmaabaGaeGOmaidacaGLOaGaayzkaaaaaa@644C@

This RMSE error has the benefit that the error of zero-imputation algorithm on any dataset is always 1, leading to a common comparison standard for comparing different algorithms on different datasets. In this paper we use the RMSE error to make the results comparable among different datasets.

## Availability

All benchmark datasets and the program of iMISS with implementation of KNN, iKNN-D, iKNN-O, LLS, iLLS-D, iLLS-O can be downloaded from the supporting website at 

## Authors' contributions

J.H. designed and implemented the algorithms, carried out the experiments, analyzed the data, and drafted the manuscript. H.L. participated in the design of the algorithm and coded the order-statistics scoring routine. M.S.W. provided scientific and technical advices and edited the manuscript. X.J.Z conceived the project, assisted in the design of the study and in drafting and editing the manuscript. All authors read and approved the final manuscript.

## References

[B1] Hoheisel JD (2006). Microarray technology: beyond transcript profiling and genotype analysis. Nat Rev Genet.

[B2] de Brevern AG, Hazout S, Malpertuy A (2004). Influence of microarrays experiments missing values on the stability of gene groups by hierarchical clustering. BMC Bioinformatics.

[B3] Bo TH, Dysvik B, Jonassen I (2004). LSimpute: accurate estimation of missing values in microarray data with least squares methods. Nucleic Acids Res.

[B4] Kim H, Golub GH, Park H (2005). Missing value estimation for DNA microarray gene expression data: local least squares imputation. Bioinformatics.

[B5] M.Scholz, F.Kaplan, C.L.Guy, J.Kopka, J.Selbig (2005). Non-linear PCA: a missing data approach. Bioinformatics.

[B6] Troyanskaya O, Cantor M, Sherlock G, Brown P, Hastie T, Tibshirani R, Botstein D, Altman RB (2001). Missing value estimation methods for DNA microarrays. Bioinformatics.

[B7] Zhou X, Wang X, Dougherty ER (2003). Missing-value estimation using linear and non-linear regression with Bayesian gene selection. Bioinformatics.

[B8] Oba S, Sato MA, Takemasa I, Monden M, Matsubara K, Ishii S (2003). A Bayesian missing value estimation method for gene expression profile data. Bioinformatics.

[B9] Sehgal MS, Gondal I, Dooley LS (2005). Collateral missing value imputation: a new robust missing value estimation algorithm for microarray data. Bioinformatics.

[B10] Wang X, Li A, Jiang Z, Feng H (2006). Missing value estimation for DNA microarray gene expression data by Support Vector Regression imputation and orthogonal coding scheme. BMC Bioinformatics.

[B11] Ouyang M, Welsh WJ, Georgopoulos P (2004). Gaussian mixture clustering and imputation of microarray data. Bioinformatics.

[B12] Jornsten R, Wang HY, Welsh WJ, Ouyang M (2005). DNA microarray data imputation and significance analysis of differential expression. Bioinformatics.

[B13] Tuikkala J, Elo L, Nevalainen OS, Aittokallio T (2006). Improving missing value estimation in microarray data with gene ontology. Bioinformatics.

[B14] (2005). Princeton SGD Lite yeast datasets. http://sgdlite.princeton.edu/download/yeast_datasets/.

[B15] DeRisi JL, Iyer VR, Brown PO (1997). Exploring the metabolic and genetic control of gene expression on a genomic scale. Science.

[B16] Ogawa N, DeRisi J, Brown PO (2000). New components of a system for phosphate accumulation and polyphosphate metabolism in Saccharomyces cerevisiae revealed by genomic expression analysis. Mol Biol Cell.

[B17] Ferea TL, Botstein D, Brown PO, Rosenzweig RF (1999). Systematic changes in gene expression patterns following adaptive evolution in yeast. Proc Natl Acad Sci U S A.

[B18] Spellman PT, Sherlock G, Zhang MQ, Iyer VR, Anders K, Eisen MB, Brown PO, Botstein D, Futcher B (1998). Comprehensive identification of cell cycle-regulated genes of the yeast Saccharomyces cerevisiae by microarray hybridization. Mol Biol Cell.

[B19] Zhou XJ, Kao MC, Huang H, Wong A, Nunez-Iglesias J, Primig M, Aparicio OM, Finch CE, Morgan TE, Wong WH (2005). Functional annotation and network reconstruction through cross-platform integration of microarray data. Nat Biotechnol.

[B20] Rhodes DR, Barrette TR, Rubin MA, Ghosh D, Chinnaiyan AM (2002). Meta-analysis of microarrays: interstudy validation of gene expression profiles reveals pathway dysregulation in prostate cancer. Cancer Res.

[B21] Choi JK, Yu U, Kim S, Yoo OJ (2003). Combining multiple microarray studies and modeling interstudy variation. Bioinformatics.

[B22] Rhodes DR, Kalyana-Sundaram S, Mahavisno V, Barrette TR, Ghosh D, Chinnaiyan AM (2005). Mining for regulatory programs in the cancer transcriptome. Nat Genet.

[B23] Lamb J RSFHLCBMRVKFSZCAPNGTREME (2003). A mechanism of cyclin D1 action encoded in the patterns of gene expression in human cancer. Cell.

[B24] Stuart JM, Segal E, Koller D, Kim SK (2003). A gene-coexpression network for global discovery of conserved genetic modules. Science.

